# Handy insights: Could online patient-reported outcome measures be used to assess hand injury rehabilitation?^✰^

**DOI:** 10.1016/j.mex.2024.103029

**Published:** 2024-11-07

**Authors:** Sten Kajitani, Nicole Liddy, David Harms, Keston Kajitani, Jason Kelly

**Affiliations:** a University College Cork, College Rd, University College, Cork, T12 K8AF; b Johns Hopkins University, Baltimore, MD, 21218, USA; c University of San Francisco, San Francisco, CA, USA; d Arizona State University, 1151 S Forest Ave, Tempe, AZ, USA

**Keywords:** Patient-reported outcome measures, Hand surgery, Michigan hand outcomes questionnaire, Hand Injury severity score, Rehabilitation, *Handy Insights: Could Online Patient-Reported Outcome Measures be used to Assess Hand Injury Rehabilitation?*

## Abstract

This protocol explores the use of online patient-reported outcome measures (PROMs) to assess recovery following hand trauma surgery. Hand injuries significantly impact patients’ functional abilities and quality of life, making PROMs crucial for tracking recovery and guiding rehabilitation. This study leverages online versions of the Michigan Hand Outcomes Questionnaire (MHQ) and Hand Injury Severity Score (HISS) to assess patient perceptions of their recovery over time.

Our prospective cohort study involves 250 participants who will complete these PROMs at four intervals post-surgery. We aim to understand how demographic factors such as age, gender, and injury severity influence recovery perceptions and outcomes. This research seeks to enhance patient care by identifying factors that influence postoperative outcomes and by tailoring rehabilitation strategies accordingly.

• **Prospective cohort study** following hand trauma surgery.
• **Online PROMs** using the MHQ and HISS.
• **Data collection at four time points**: baseline, 2–4 weeks, 6–8 weeks, and 10–14 weeks post-surgery.

By making these tools accessible online, this protocol aims to improve patient engagement, increase response rates, and provide valuable insights to guide personalized rehabilitation strategies.

## Graphical abstract

**Figure:**
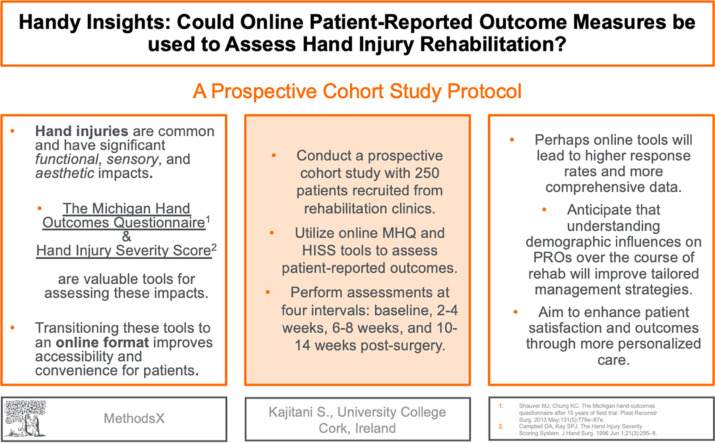

Specifications table

**Table:** 

Subject area:	Medicine and Dentistry
More specific subject area:	*Hand Trauma Surgery*
Name of your method:	*Handy Insights: Could Online Patient-Reported Outcome Measures be used to Assess Hand Injury Rehabilitation?*
Name and reference of original method:	*None*
Resource availability:	*None*

## Background

Hands play a crucial role in our daily lives, contributing to functional, sensory, and aesthetic aspects. However, they are also particularly vulnerable to various injuries. In the United States, hand injuries are a significant public health concern. From 1990 to 2009, approximately 16.4 million children under 18 were treated for hand injuries in emergency departments, averaging 818,688 injuries annually [1]. More recently, between 2009 and 2018, the annual incidence rates for finger, hand, and wrist injuries were 450, 264, and 182 per 100,000 people, respectively, amounting to roughly 2.6 million emergency department visits each year [2]. Acute hand injuries are not only common but also costly, with substantial economic implications due to medical care, rehabilitation, and lost productivity. Annually, hand and wrist injuries account for approximately $740 million, ranking them as the most expensive injury types. Notably, productivity costs make up 56% of the total economic burden, surpassing direct healthcare expenses [3].

Measuring patient-reported outcomes (PROs) is vital because these metrics capture the patients' perspectives on their health, recovery, and overall quality of life [4]. Negative PROs can significantly impact patients' adherence to postoperative therapy, coping strategies, and clinical outcomes [4]. Traditionally, the primary focus of hand surgeons has been on restoring function [5]. However, more recent literature highlights the importance of addressing both functional and aesthetic outcomes [6]. Understanding how patients prioritize these outcomes is essential for optimizing surgical interventions and rehabilitation strategies.

The introduction of online versions of the Michigan Hand Outcomes Questionnaire (MHQ) and the Hand Injury Severity Score (HISS) calculator represents a significant advancement in making these tools more accessible [7]. Online formats enhance convenience for patients, allowing them to complete assessments at their own pace and in their preferred setting [8]. This protocol hopes to provide an accessible online version of the MHQ and an online HISS calculator, facilitating easier data collection and potentially improving response rates and data quality.

It is understood that hand injuries have a profound impact on patients' quality of life, influencing both their functional capabilities and their psychological well-being [9]. The MHQ is a validated tool used to assess various dimensions of hand health, including overall function, activities of daily living (ADLs), pain, work performance, aesthetics, and patient satisfaction [10,11]. Additionally, the HISS is a reliable measure of injury severity that can be used to predict outcomes and guide treatment [7,12].

However, we do not fully understand how demographic factors such as age, gender, race, education, and income influence PROs after hand trauma surgery [13,14]. There is also limited data on how patients' attitudes towards their outcomes change over the course of rehabilitation and how these attitudes impact their overall recovery and satisfaction [15,16]. Our primary question was, "What are the factors that affect patient perceptions and how do patients’ attitudes towards their outcomes change over the course of rehab?"

## Method details

### Aims and objectives

Our study aims to assess PROs following hand trauma surgery, enhance surgeons’ knowledge of PROs to facilitate treatment interventions that mitigate any negative PRO, and identify the prevalence of participants who prioritize hand function restoration over aesthetics, or vice versa. We also aim to determine if participants would accept a less aesthetic outcome for a surgery more focused on functional restoration after a traumatic hand injury depending on their stage of recovery [15,16]. Furthermore, we seek to identify demographic factors such as age, gender, and severity of trauma that may impact patients' acceptance of a less aesthetic outcome after surgery [13,14].

By characterizing a population undergoing hand trauma surgery and conducting a survey-based prospective cohort study using the Michigan Hand Outcomes Questionnaire (MHQ), we aim to assess the factors that affect PROs following hand trauma surgery [12,13]. Perhaps understanding PROs can enhance patient satisfaction and positively influence a patient's overall experience of their care and perception of their surgeon [17]. By enhancing surgeons' knowledge of PROs, this study aims to facilitate treatment interventions that mitigate any negative PRO [18]. This project aspires to explore PROs following hand trauma surgery with the hopes of providing valuable insights that can enhance patient care and optimize rehabilitation strategies.

### Study design

An experimental prospective cohort study with four-time interval assessments post-surgery will be carried out, following the recommendations of CONSORT (Consolidated Standards of Reporting Trials) to assess the effectiveness of the intervention [19].

### Subjects

Subjects will be selected from among patients with hand trauma who have undergone surgery.

### Inclusion and exclusion criteria

- **Inclusion:**
- 18 years old or over
- Willingness to fill out the online questionnaires
- Ability to understand informed-consent process and to document informed consent before completion of any study-related procedure
  Ability to read, comprehend, and complete patient-reported outcome measures in English
- Patients that have undergone hand trauma surgery.
- **Exclusion:**
- Patients with dementia or other cognitive impairment.
- Pregnant women.
- Prisoners.
- Patients attending the outpatient department for reasons other than post-operative follow-up for hand surgery.

### Sample size calculation

The estimated sample size required for adequate power is 250. This was calculated using the maximum number of patients who attended the outpatient department following hand trauma surgery between September 2023 and March 2024, which is estimated to be 600. A 95% confidence level and a margin of error of 5% were applied to this population, yielding a sample size of 235. Considering a 5% attrition rate, the final sample size was rounded up to 250.

### Recruitment

Subjects will be recruited from hand clinics where they have started a recovery program after hand surgery. They will be invited to take part in the study immediately after their appointment. They will be informed about the aims of the study and asked for informed consent.

### Procedures

Subjects will receive an invitation letter, an information sheet, and a consent form. Upon consent, the investigator will record the subjects’ injuries on the HISS data acquisition sheet, which will then be used to calculate the Hand Injury Severity Score as a potential variable associated with PROs following hand surgery (Appendix 1–2) [7,12].

The subjects will then complete the baseline questionnaire via a QR-code on the information sheet (Appendix 3). The baseline questionnaire will include:

A General Information Section

• Identifiers: Patients will be asked to provide their name and email as part of the general information section. These will be used to cross-reference their baseline questionnaire with operative notes and the following questionnaires.
  Demographic Information: This section of the baseline questionnaire collects essential demographic data from subjects (ie. Ethnicity, Race, Gender, Age, Hand Dominance, Occupation, Education Level, Income. Understanding the demographic background of patients helps in analyzing how various factors influence PROs after hand surgery [13,14].

An Online Copy of the MHQ

• To ensure the validity and reliability of the research findings, the study has been licensed to use a quantitative Michigan Hand Outcomes Questionnaire, sourced and validated by the literature, as a patient reported outcome measure (PROM) for PRO following hand surgery (Appendix 4) [10,11].
• Customized to a Google form format, “the Michigan Hand Outcomes Questionnaire (MHQ) is a tool used to assess patients with hand disorders through the measurement of 6 health domains: overall hand function, activities of daily living (ADLs), pain, work performance, aesthetics, and patient satisfaction” [10,11]. The MHQ was adjusted slightly to more fluently translate to a target population [20]. Specifically questions were added regarding how many physical rehabilitation sessions the patient has attended and how their doctor/therapist summarizes their progress. The survey also included a non-required question regarding whether their case was currently pending in court as a potential variable that affects PROs [21].

The subjects will then be contacted via email for follow-up questionnaires at three subsequent time intervals: 2–4 weeks, 6–8 weeks, and 10–14 weeks post-surgery (Appendix 5–7).

### Data protection

The data will not be accessible to anyone outside of the research team. Their name and email will be removed from the Excel file as soon as they are no longer required. Codes will be used to pseudonymise the data and will be destroyed once cross-referencing requirements have ended. The information provided will be used solely for research purposes and will remain confidential.

### PROM description

In selecting a Patient-Reported Outcome Measure (PROM), it is crucial to consider potential ceiling and floor effects to ensure the chosen outcome measures provide adequate precision and enable effective patient stratification. Shortened questionnaires like SF12 and Brief-MHQ, which use only one or two items to assess each domain, have been developed to address issues associated with longer questionnaires, such as incomplete data, lower response rates, and reduced quality of results due to responder fatigue and loss to follow-up [22]. However, there is limited research on the use of these shortened questionnaires to evaluate PROs following hand surgery.

In contrast, longer questionnaires like the MHQ can capture the multidimensional aspects of a complex disease such as a hand injury. Rasch analysis has demonstrated that the MHQ is a reliable and valid measure for evaluating hand outcomes in older adults following treatment for distal radius fractures. This rigorous approach to questionnaire validation has been used to assess the psychometric properties of the MHQ in various conditions. However, results from Rasch analysis suggest that MHQ scores should be interpreted in a condition-specific manner, with a focus on individual domain scores rather than the summary MHQ score [23]. To address these issues, this study may employ Item Response Theory (IRT) via Computerized Adaptive Testing (CAT) to model respondents' responses according to their level of function or ability.

The use of an online formatted questionnaire could significantly enhance accessibility and convenience for patients. By allowing patients to complete assessments at their own pace and in their preferred setting, perhaps online tools like the MHQ and HISS can lead to higher response rates and more comprehensive data [8]. This accessibility ensures that a broader range of patients can participate in the study, thereby improving the quality and reliability of the collected data.

### Evaluation

Data from the MHQ and HISS will be collected at four-time intervals post-surgery: baseline (day 1 post-operation), 2–4 weeks, 6–8 weeks, and 10–14 weeks post-surgery. The information gathered will include patient demographics, hand injury severity, and PROs related to overall hand function, activities of daily living (ADLs), pain, work performance, aesthetics, and patient satisfaction [10,11].

### Statistical methods

Data obtained from the questionnaires will be transferred and exported to a password-protected excel file and then into IBM SPSS Statistics version 27.0 for analysis. Values related to the MHQ will be reported as mean ± standard error. Multiple linear regression analysis will be performed to determine whether categorical variables (e.g., patient race, gender, injury severity) and numerical variables (e.g., patient age) predict PRO following hand surgery, allowing assessment of the relative importance of various demographic and clinical factors in predicting MHQ scores [24]. Assumptions of normality will be checked using Shapiro-Wilk tests and Q-Q plots, homoscedasticity using Levene's test, and multicollinearity using Variance Inflation Factors [25]. Pearson's correlation will be used to examine relationships between continuous variables, while independent samples *t*-tests will compare means between binary categorical variables. For categorical variables with more than two groups (e.g., injury severity levels based on HISS scores), one-way ANOVA will be employed to compare group means [26]. The HISS will be analyzed both as a continuous variable in regression analyses and as a categorical variable (Minor, Moderate, Severe, Major). We will use ANOVA to compare MHQ scores between these groups and examine how patients with different injury severities show different patterns of recovery over the four time points. This will also allow us to analyze the data within the categories for a more meaningful interpretation. To analyze changes in MHQ scores across the four time points (baseline, 2–4 weeks, 6–8 weeks, and 10–14 weeks post-surgery), repeated measures ANOVA will be conducted, with post-hoc tests using Bonferroni correction for multiple comparisons when significant differences are found [27]. Effect sizes will be reported using Cohen's d for *t*-tests and partial eta-squared for ANOVA to provide information about the magnitude of observed effects [28]. Missing data will be handled using multiple imputation techniques to preserve statistical power and reduce bias [29]. A power analysis using G*Power 3.1 indicated that to detect a medium effect size (f² = 0.15) in multiple regression with up to 10 predictors, assuming α = 0.05 and 80% power, a minimum sample size of 118 is required [30]. Our planned sample of 250 exceeds this requirement, allowing for potential attrition. A significance level of *p* < 0.05 will be considered for all analyses.

## Method validation

In this study, we aim to explore patient-reported outcomes (PROs) following hand trauma surgery by examining the factors that influence patient perceptions and how these perceptions evolve during rehabilitation. Introducing online versions of the MHQ and the HISS calculator might make these tools more accessible and convenient for patients, which could enhance data collection and improve rehabilitation strategies [8].

In selecting a Patient-Reported Outcome Measure (PROM), it is crucial to consider potential ceiling and floor effects to ensure the chosen outcome measures provide adequate precision and enable effective patient stratification. Shortened questionnaires like SF12 and Brief-MHQ, which use only one or two items to assess each domain, have been developed to address issues associated with longer questionnaires, such as incomplete data, lower response rates, and reduced quality of results due to responder fatigue and loss to follow-up [22]. However, there is limited research on the use of these shortened questionnaires to evaluate PROs following hand surgery.

In contrast, longer questionnaires like the MHQ can capture the multidimensional aspects of a complex disease such as a hand injury. Rasch analysis has demonstrated that the MHQ is a reliable and valid measure for evaluating hand outcomes in older adults following treatment for distal radius fractures. This rigorous approach to questionnaire validation has been used to assess the psychometric properties of the MHQ in various conditions. However, results from Rasch analysis suggest that MHQ scores should be interpreted in a condition-specific manner, with a focus on individual domain scores rather than the summary MHQ score [23]. To address these issues, this study may employ Item Response Theory (IRT) via Computerized Adaptive Testing (CAT) to model respondents' responses according to their level of function or ability.

The use of an online formatted questionnaire could significantly enhance accessibility and convenience for patients. By allowing patients to complete assessments at their own pace and in their preferred setting, perhaps online tools like the MHQ and HISS can lead to higher response rates and more comprehensive data [8]. This accessibility ensures that a broader range of patients can participate in the study, thereby improving the quality and reliability of the collected data.

Hand injuries can severely impact functional capabilities and psychological well-being, making it crucial to measure PROs [9,18]. Demographic variables such as age, gender, race, education, and income are known to influence these outcomes [13,14]. By understanding these factors, we might be able to tailor surgical and rehabilitation strategies to meet the diverse needs of different patient groups more effectively.

The online MHQ and HISS tools are expected to provide a user-friendly way for patients to complete assessments, potentially leading to higher response rates and more complete data [8]. This convenience may allow patients to engage with the assessments at their own pace and setting, resulting in more accurate and comprehensive data on PROs.

This research will build on previous studies by demonstrating the feasibility and effectiveness of online PRO tools in a clinical setting, improving on traditional paper-based questionnaires [8]. The shift to an online format could represent a significant improvement in accessibility and ease of use, leading to better data collection and more meaningful insights.

Understanding the factors influencing PROs might significantly impact clinical practice. Perhaps younger patients may benefit from detailed discussions about cosmetic outcomes, while older patients might prioritize functional recovery and pain management [5,6]. Tailoring communication and treatment strategies to specific patient needs could enhance satisfaction and outcomes [17].

## Limitations

While our methodology using online patient-reported outcome measures (PROMs) offers several advantages, there are notable limitations that may affect its applicability and effectiveness in certain populations or situations. Recognizing these limitations is essential for accurately interpreting the study's results and for addressing potential challenges in future research.

1. Digital Access Barriers: One of the primary limitations of this method is the assumption that all participants have access to digital devices, such as smartphones, tablets, or computers, and a reliable internet connection. Individuals without access to these technologies may be excluded from the study or experience difficulty completing the assessments. This could disproportionately affect participants from lower socioeconomic backgrounds, older populations, or those in rural or underserved areas, where internet access may be limited. Additionally, certain participants may not be comfortable using digital platforms, further limiting the generalizability of the findings.
2. Literacy and Health Literacy: Another limitation is the reliance on participants' ability to read and comprehend the online questionnaires. While the MHQ and HISS are designed to be straightforward, patients with low literacy or health literacy may struggle to complete the assessments accurately. This issue could be exacerbated for patients for whom English is not their first language, particularly if translated versions of the tools are not available.
3. Cognitive or Physical Impairments: Some participants with hand trauma may have additional cognitive or physical impairments that make it difficult to complete the online assessments. Cognitive impairments, such as dementia or post-traumatic stress, may hinder patients' ability to understand or follow through with the survey questions. Likewise, severe hand injuries may limit a patient's ability to effectively use digital devices, especially if they have restricted dexterity, making it challenging to complete the assessments without assistance.
4. Attrition and Follow-up Challenges: This study involves multiple follow-up points over several weeks post-surgery (2–4 weeks, 6–8 weeks, and 10–14 weeks), which can be challenging for some participants. Loss to follow-up, common in longitudinal studies, may occur if patients fail to complete subsequent questionnaires, whether due to waning motivation, forgetfulness, or external factors such as changes in health status or lack of internet access at later stages of recovery. Attrition could lead to incomplete data and reduce the statistical power of the study, potentially biasing the results.
5. Exclusion of Certain Populations: By excluding patients with cognitive impairments, illiteracy, or those without access to digital tools, we may inadvertently overlook an important segment of the population. These excluded patients may have unique rehabilitation challenges and outcomes that are not captured in the study, limiting the generalizability of the results to the broader population of hand trauma patients.
6. Technological Issues: Lastly, technical difficulties with the online platform could affect data collection. Participants may encounter problems accessing the online forms or submitting their responses, particularly if the survey platform is incompatible with their device or if they experience connectivity issues. These technological barriers could hinder participation and affect the completeness of the data.

In summary, while the use of online PROMs provides an accessible and efficient way to assess patient-reported outcomes following hand trauma surgery, it is important to acknowledge that digital access, literacy, physical and cognitive ability, follow-up retention, and technological issues may limit the method's applicability across diverse patient populations. Addressing these challenges in future research could improve inclusivity and ensure broader applicability of the findings.

## Ethics statements

This study protocol was reviewed and approved by the Clinical Research Ethics Committee (CREC) of the Cork Teaching Hospitals (Approval No: ECM 6 (e)). All data will be anonymized and stored securely, adhering to GDPR guidelines. Participation in the study is voluntary, after written informed consent from all subjects. Subjects’ consent will be obtained before data collection, and all steps will comply with the Data Protection Act 2018.

## CRediT authorship contribution statement

**Sten Kajitani:** Conceptualization, Methodology, Writing – original draft, Investigation. **Nicole Liddy:** Writing – review & editing. **David Harms:** Writing – review & editing. **Keston Kajitani:** Writing – review & editing. **Jason Kelly:** Validation, Supervision.

## Declaration of competing interest

The authors declare that they have no known competing financial interests or personal relationships that could have appeared to influence the work reported in this paper.

## Data Availability

The data that has been used is confidential.

## Appendix Supplementary materials

Image, application 1

Image, application 2

Image, application 3

Image, application 4

Image, application 5

Image, application 6

Image, application 7

Image, application 8

Image, application 9
