# Supplementary material for: Handy insights: Could online patient-reported outcome measures be used to assess hand injury rehabilitation?
Source: MethodsX. 2024 Nov 7;13:103029. doi: 10.1016/j.mex.2024.103029 (PMC11600655; doi:10.1016/j.mex.2024.103029)

***INSERT WATERMARK***

**Participant Invitation Letter**

My name is _________________ . I am a _________________ at _________________ **.**

The registered title of my project is “*Assessing Patient Reported Outcomes Following Hand Trauma Surgery in* _________________  *: A Prospective Cohort Study*”

My supervisor is _________________ , Consultant in _________________ surgery at _________________ Hospital

We have faced challenges with recruitment and follow up for our study so far. As a result, each questionnaire entry will be entered in a raffle for a _________________ . I would greatly appreciate your participation because there is evidence that understanding patient reported outcomes can inform more targeted interventions to improve patient satisfaction and their quality of life. However, there is currently limited data on patient reported outcomes in the _________________ Surgery department at _________________ Hospital (...) in _________________ . Your participation will help doctors and surgeons understand how people truly feel about their surgical scars so actions can be taken to help.

Should you wish to take part you will be asked to complete a questionnaire online. It consists of a general information section and a validated hand surgery outcome questionnaire. There will also be three follow up questionnaires conducted over an emailed link 1 month, 2 months, and 3 months from now. These will be slightly shorter questionnaires, as the general information section will not be included. These questionnaires should take about 15 minutes to complete each time, and your answers will be entirely anonymous to anyone outside of the research team.

Your participation is voluntary and you are free to withdraw at any time.

Should you decide to participate, the information gathered as part of this study will be used only in this study. No identifiable information about you will be used in the analysis.

Thank you for your help in this matter.

***Sign:*** __________________________________

*If you are still interested in taking part please read and sign the participant consent form on the back of this page.*

***INSERT WATERMARK***

**Participant Consent Form**

Study Title: *Assessing Patient Reported Outcomes Following Hand Trauma Surgery in …: A Prospective Cohort Study*

Primary Investigator: _____________________________________________________

First Author: ____________________________________________________________

Email Address: __________________________________________________________

By signing this document, I agree to take part in the above named study. I have read the Participant Information Leaflet and I am fully aware of what will be required of me.

I understand that I am participating on a voluntary basis and may withdraw from this study at any time and that my withdrawal will not result in any repercussions.

I acknowledge that by participating, I understand that the information I give may be published in a research journal.

I agree to information taken from my chart to be used in this study (please **tick** the box) ☐

I agree to be contacted by the email I provide in 1, 2, and 3 month’s time for the follow up questionnaires (please **tick** the box) ☐

Participant Name (PRINT) __________________________

Participant Signature ______________________________ Date: _______________

**After signing this consent form, please open the camera app on your Smartphone, and point it at the following QR code to Scan and access the baseline questionnaire:*

**If you do not have a QR scanner, please provide your email and we will email you the questionnaires: _______________________________________*

**Please provide your phone number if you would like help filling out the questionnaire: _______________________________________*


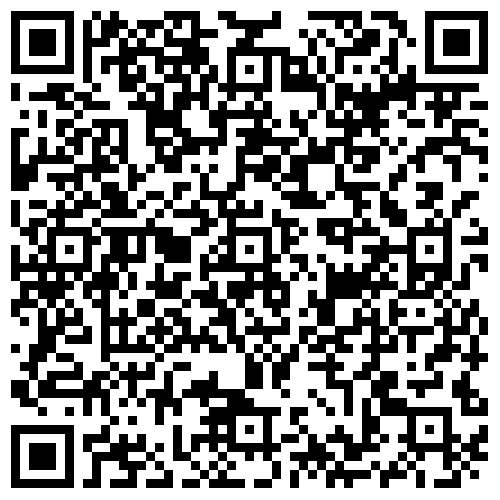

Supplement: Supplementary file 2 [file mmc2.docx]
