# Supplementary material for: Handy insights: Could online patient-reported outcome measures be used to assess hand injury rehabilitation?
Source: MethodsX. 2024 Nov 7;13:103029. doi: 10.1016/j.mex.2024.103029 (PMC11600655; doi:10.1016/j.mex.2024.103029)
Supplement: Supplementary file 3 [file mmc3.pdf]

Permission to Make Copies and Edit

If you would like permission to edit and make copies of this questionnaire, please provide your email, name, and academic affiliation. Requests must come from a valid academic domain email address (.edu or equivalent) and must include a description of your study and research in the purposes section of this form. You must also prove that you have the MHQ Academic and Research Education Use License, which can be accessed via this link: MHQ ACADEMIC AND RESEARCH EDUCATION USE LICENSE APPLICATION

Failure to provide this information will result in the denial of your request.

Upon completion of section 1 and submission of this form, our team will be notified, and we will email you a copy of the baseline MHQ, MHQ 2-4, and the Hand Injury Severity Score Calculator, with permission to edit for your purposes.

* Indicates required question

1. Email *

____

2. Name of Investigator *

____

3. Name of Academic Affiliation *

____

4. Purpose for using this version of MHQ *

____
____
____
____
____

5. Proof of MHQ Academic and Research Education Use License (PDF) *

Files submitted:

Questionnaire 1 (Baseline)

Study Title: *Assessing Patient Reported Outcomes Following Hand Trauma Surgery: A Prospective Cohort Study*

Chief Investigator:

Medical Student:
Email Address:

Dear Participant,
I greatly appreciate your participation because there is evidence that understanding patient attitudes towards their postoperative outcomes can inform more targeted interventions to improve patient satisfaction and their quality of life. However, there is currently limited data on patient attitudes towards post-surgical outcomes in the ... Hospital in ... . Your participation will help doctors and surgeons understand how people truly feel about their surgical scars so actions can be taken to help.

Should you wish to take part you will be asked to complete a questionnaire online. It consists of a general information section and a validated hand surgery outcome questionnaire. There will also be three follow up questionnaires conducted over an emailed link 1 month, 2 months, and 3 months from now. These will be slightly shorter questionnaires, as the general information section will not be included. These questionnaires should take between 15 minutes to complete each time, and your answers will be entirely anonymous to anyone outside of the research team.

Your participation is voluntary and you are free to withdraw at any time.
Should you decide to participate, the information gathered as part of this study will be used only in this study. No identifiable information about you will be used in the analysis.

6. Please provide your name (First Last)

____

7. Please provide your phone number (if applicable):

____

8. Please review the following: Participant Information Leaflet and Data Protection Notice

***If you have any questions**, or need help filling out the form, remember that you can email the chief investigator, **...**, at **...**, or phone them at **...**

Check all that apply.

☐ By ticking the box on this document, I agree to take part in the above named study. I have read the Participant Information Leaflet and Data Protection Notice, and I am fully aware of what will be required of me and how my Data will be protected. I understand that I am participating on a voluntary basis and may withdraw from this study at any time and that my withdrawal will not result in any repercussions. I acknowledge that by participating, I understand that the information I give may be published in a research journal. I acknowledge that by participating, I understand that the information I give may be published in a research journal.

MICHIGAN HAND OUTCOMES QUESTIONNAIRE (MHQ)

Instructions: This survey asks for your views about your hands and your health. This information will help keep track of how you feel and how well you are able to do your usual activities.

- Answer **EVERY** question, even if you do not experience any problems with the hand and/or wrist. If you are unsure about how to answer a question, please give the best answer you can.

Please provide the following information about yourself. (Please circle one answer for each question).

9. 1. Are you right-handed or left-handed?

Mark only one oval.

- ◯ Right-handed
- ◯ Left-handed
- ◯ Both

10. 2. What is your ethnic background?

Mark only one oval.

- Hispanic or Latino/a
- Not Hispanic or Latino/a

11. 3. What is your racial background?

Check all that apply.

- American Indian or Alaskan Native
- Asian
- Native Hawaiian or Other Pacific Islander
- Black or African
- White
- Other: ______________________

12. 4. a. Please describe the type of job you did **before** you injured your hands(s).

____

13. 4. b. Please describe the type of job you are doing **now**.

____

14. 5. How many days after your surgery did you return to work/plan to start work?

____

15. 6. How long after your surgery did you/plan to return to the same job you were doing before your injury?

____

16. 7. What is your gender?

Mark only one oval.

- Male
- Female

17. 8. What is the highest level of education you received?

Mark only one oval.

- Less than high school graduate
- High school graduate or GED
- Vocational/techinical school
- Some college or Associate degree
- College graduate
- Professional or graduate school

18. 9. What is your approximate family income including wages, disability payment, retirement income and welfare?

Mark only one oval.

- a. Less than 10,000
- b. $10,000 - €19,999
- c. $20,000 - $29,999
- d. $30,000 - $39,999
- e. $40,000 - $49,999
- f. $50,000 - $59,999
- g. $60,000 - $69,999
- h. More than $70,000

19. How old are you? *

MICHIGAN HAND OUTCOMES QUESTIONNAIRE (MHQ)

Instructions: This survey asks for your views about your hands and your health. This information will help keep track of how you feel and how well you are able to do your usual activities.

- Answer **EVERY** question, even if you do not experience any problems with the hand and/or wrist. If you are unsure about how to answer a question, please give the best answer you can.

I. The following questions refer to the function of your hand(s)/wrist(s) ***during the past week.***

A. The following questions refer to your ***right*** hand/wrist. (Please circle one answer for each question).

20. 1. Overall, how well did your ***right*** hand work?

Mark only one oval.

- Very Good
- Good
- Fair
- Poor
- Very Poor
- N/A

21. 2. How well did your ***right*** fingers move?

Mark only one oval.

- Very Good
- Good
- Fair
- Poor
- Very Poor
- N/A

22. 3. How well did your ***right*** wrist move?

Mark only one oval.

- Very Good
- Good
- Fair
- Poor
- Very Poor
- N/A

23. 4. How was the strength in your ***right*** hand?

Mark only one oval.

- Very Good
- Good
- Fair
- Poor
- Very Poor
- N/A

24. 5. How was the sensation (feeling) in your ***right*** hand?

Mark only one oval.

- Very Good
- Good
- Fair
- Poor
- Very Poor
- N/A

MICHIGAN HAND OUTCOMES QUESTIONNAIRE (MHQ)

Instructions: This survey asks for your views about your hands and your health. This information will help keep track of how you feel and how well you are able to do your usual activities.

- Answer **EVERY** question, even if you do not experience any problems with the hand and/or wrist. If you are unsure about how to answer a question, please give the best answer you can.

I. The following questions refer to the function of your hand(s)/wrist(s) ***during the past week.***
B. The following questions refer to your ***left*** hand/wrist. (Please circle one answer for each question).

25. 1. Overall, how well did your ***left*** hand work?

Mark only one oval.

- Very Good
- Good
- Fair
- Poor
- Very Poor
- N/A

26. 2. How well did your ***left*** fingers move?

Mark only one oval.

- Very Good
- Good
- Fair
- Poor
- Very Poor
- N/A

27. 3. How well did your ***left*** wrist move?

Mark only one oval.

- Very Good
- Good
- Fair
- Poor
- Very Poor
- N/A

28. 4. How was the strength in your ***left*** hand?

Mark only one oval.

- Very Good
- Good
- Fair
- Poor
- Very Poor
- N/A

29. 5. How was the sensation (feeling) in your ***left*** hand?

Mark only one oval.

- Very Good
- Good
- Fair
- Poor
- Very Poor
- N/A

MICHIGAN HAND OUTCOMES QUESTIONNAIRE (MHQ)

Instructions: This survey asks for your views about your hands and your health. This information will help keep track of how you feel and how well you are able to do your usual activities.

- Answer **EVERY** question, even if you do not experience any problems with the hand and/or wrist. If you are unsure about how to answer a question, please give the best answer you can.

II. The following questions refer to the ability of your hand(s) to do certain tasks ***during the past week***. (Please circle one answer for each question). If you do not do a certain task, please estimate the difficulty with which you would have in performing it.
A. How difficult was it for you to perform the following activities using your ***right hand***?

30. 1. Turn a door knob

Mark only one oval.

- Not at All Difficult
- A Little Difficult
- Somewhat Difficult
- Moderately Difficult
- Very Difficult
- N/A

31. 2. Pick up a coin

Mark only one oval.

- Not at All Difficult
- A Little Difficult
- Somewhat Difficult
- Moderately Difficult
- Very Difficult
- N/A

32. 3. Hold a glass of water

Mark only one oval.

- Not at All Difficult
- A Little Difficult
- Somewhat Difficult
- Moderately Difficult
- Very Difficult
- N/A

33. 4. Turn a key in a lock

Mark only one oval.

- Not at All Difficult
- A Little Difficult
- Somewhat Difficult
- Moderately Difficult
- Very Difficult
- N/A

34. 5. Hold a frying pan

Mark only one oval.

- Not at All Difficult
- A Little Difficult
- Somewhat Difficult
- Moderately Difficult
- Very Difficult
- N/A

MICHIGAN HAND OUTCOMES QUESTIONNAIRE (MHQ)

Instructions: This survey asks for your views about your hands and your health. This information will help keep track of how you feel and how well you are able to do your usual activities.

- Answer **EVERY** question, even if you do not experience any problems with the hand and/or wrist. If you are unsure about how to answer a question, please give the best answer you can.

II. The following questions refer to the ability of your hand(s) to do certain tasks ***during the past week***. (Please circle one answer for each question). If you do not do a certain task, please estimate the difficulty with which you would have in performing it.

B. How difficult was it for you to perform the following activities using your ***left hand***?

35. 1. Turn a door knob

Mark only one oval.

- Not at All Difficult
- A Little Difficult
- Somewhat Difficult
- Moderately Difficult
- Very Difficult
- N/A

36. 2. Pick up a coin

Mark only one oval.

- Not at All Difficult
- A Little Difficult
- Somewhat Difficult
- Moderately Difficult
- Very Difficult
- N/A

37. 3. Hold a glass of water

Mark only one oval.

- Not at All Difficult
- A Little Difficult
- Somewhat Difficult
- Moderately Difficult
- Very Difficult
- N/A

38. 4. Turn a key in a lock

Mark only one oval.

- Not at All Difficult
- A Little Difficult
- Somewhat Difficult
- Moderately Difficult
- Very Difficult
- N/A

39. 5. Hold a frying pan

Mark only one oval.

- Not at All Difficult
- A Little Difficult
- Somewhat Difficult
- Moderately Difficult
- Very Difficult
- N/A

MICHIGAN HAND OUTCOMES QUESTIONNAIRE (MHQ)

Instructions: This survey asks for your views about your hands and your health. This information will help keep track of how you feel and how well you are able to do your usual activities.

- Answer **EVERY** question, even if you do not experience any problems with the hand and/or wrist. If you are unsure about how to answer a question, please give the best answer you can.

II. The following questions refer to the ability of your hand(s) to do certain tasks ***during the past week***. (Please circle one answer for each question). If you do not do a certain task, please estimate the difficulty with which you would have in performing it.
C. How difficult was it for you to perform the following activities using ***both of your hands?***

40. 1. Open a jar

Mark only one oval.

- Not at All Difficult
- A Little Difficult
- Somewhat Difficult
- Moderately Difficult
- Very Difficult
- N/A

41. 2. Button a shirt/blouse

Mark only one oval.

- Not at All Difficult
- A Little Difficult
- Somewhat Difficult
- Moderately Difficult
- Very Difficult
- N/A

42. 3. Eat with a knife/fork

Mark only one oval.

- Not at All Difficult
- A Little Difficult
- Somewhat Difficult
- Moderately Difficult
- Very Difficult
- N/A

43. 4. Carry a grocery bag

Mark only one oval.

- Not at All Difficult
- A Little Difficult
- Somewhat Difficult
- Moderately Difficult
- Very Difficult
- N/A

44. 5. Wash dishes

Mark only one oval.

- Not at All Difficult
- A Little Difficult
- Somewhat Difficult
- Moderately Difficult
- Very Difficult
- N/A

45. 6. Wash your hair

Mark only one oval.

- Not at All Difficult
- A Little Difficult
- Somewhat Difficult
- Moderately Difficult
- Very Difficult
- N/A

46. 7. Tie shoelaces/knots

Mark only one oval.

- Not at All Difficult
- A Little Difficult
- Somewhat Difficult
- Moderately Difficult
- Very Difficult
- N/A

MICHIGAN HAND OUTCOMES QUESTIONNAIRE (MHQ)

Instructions: This survey asks for your views about your hands and your health. This information will help keep track of how you feel and how well you are able to do your usual activities.

- Answer **EVERY** question, even if you do not experience any problems with the hand and/or wrist. If you are unsure about how to answer a question, please give the best answer you can.

III. The following questions refer to how you did in your ***normal work*** (including both housework and school work) during the ***past four weeks.*** (Please circle one answer for each question).

47. 1. How often were you unable to do your work because of problems with your hand(s)/wrist(s)?

Mark only one oval.

- Always
- Often
- Sometimes
- Rarely
- Never
- N/A

48. 2. How often did you have to shorten your work day because of problems with your hand(s)/ wrist(s)?

Mark only one oval.

- Always
- Often
- Sometimes
- Rarely
- Never
- N/A

49. 3. How often did you have to take it easy at your work because of problems with your hand(s)/ wrist(s)?

Mark only one oval.

- Always
- Often
- Sometimes
- Rarely
- Never
- N/A

50. 4. How often did you accomplish less in your work because of problems with your hand(s)/ wrist(s)?

Mark only one oval.

- Always
- Often
- Sometimes
- Rarely
- Never
- N/A

51. 5. How often did you take longer to do the tasks in your work because of problems with your hand(s)/ wrist(s)?

Mark only one oval.

- Always
- Often
- Sometimes
- Rarely
- Never
- N/A

MICHIGAN HAND OUTCOMES QUESTIONNAIRE (MHQ)

Instructions: This survey asks for your views about your hands and your health. This information will help keep track of how you feel and how well you are able to do your usual activities.

- Answer **EVERY** question, even if you do not experience any problems with the hand and/or wrist. If you are unsure about how to answer a question, please give the best answer you can.

IV. The following questions refer to how much ***pain*** you had in your hand(s)/wrist(s) ***during the past week.*** (Please circle one answer for each question).

A. The following questions refer to **pain** in your **right** hand/wrist.

52. 1. How often did you have pain in your ***right*** hand/wrist?

Mark only one oval.

- Always
- Often
- Sometimes
- Rarely
- Never

MICHIGAN HAND OUTCOMES QUESTIONNAIRE (MHQ)

Instructions: This survey asks for your views about your hands and your health. This information will help keep track of how you feel and how well you are able to do your usual activities.

- Answer **EVERY** question, even if you do not experience any problems with the hand and/or wrist. If you are unsure about how to answer a question, please give the best answer you can.

IV. The following questions refer to how much ***pain*** you had in your hand(s)/wrist(s) ***during the past week.*** (Please circle one answer for each question).

A. The following questions refer to **pain** in your **right** hand/wrist.

53. 2. Please describe the pain you had in your ***right*** hand/wrist

Mark only one oval.

- Very Mild
- Mild
- Moderate
- Severe
- Very Severe
- N/A

54. 3. How often did the pain in your ***right*** hand/wrist interfere with your sleep?

Mark only one oval.

- Always
- Often
- Sometimes
- Rarely
- Never
- N/A

55. 4. How often did the pain in your ***right*** hand/wrist interfere with your daily activities (such as eating or bathing)?

Mark only one oval.

- Always
- Often
- Sometimes
- Rarely
- Never
- N/A

56. 5. How often did the pain in your ***right*** hand/wrist make you unhappy?

Mark only one oval.

- Always
- Often
- Sometimes
- Rarely
- Never
- N/A

MICHIGAN HAND OUTCOMES QUESTIONNAIRE (MHQ)

Instructions: This survey asks for your views about your hands and your health. This information will help keep track of how you feel and how well you are able to do your usual activities.

- Answer **EVERY** question, even if you do not experience any problems with the hand and/or wrist. If you are unsure about how to answer a question, please give the best answer you can.

IV. The following questions refer to how much ***pain*** you had in your hand(s)/wrist(s) ***during the past week.*** (Please circle one answer for each question).
B. The following questions refer to **pain** in your **left** hand/wrist.

57. 1. How often did you have pain in your ***left*** hand/wrist?

Mark only one oval.

- Always
- Often
- Sometimes
- Rarely
- Never

MICHIGAN HAND OUTCOMES QUESTIONNAIRE (MHQ)

Instructions: This survey asks for your views about your hands and your health. This information will help keep track of how you feel and how well you are able to do your usual activities.

- Answer **EVERY** question, even if you do not experience any problems with the hand and/or wrist. If you are unsure about how to answer a question, please give the best answer you can.

58. 2. Please describe the pain you had in your ***left*** hand/wrist

Mark only one oval.

- Very Mild
- Mild
- Moderate
- Severe
- Very Severe
- N/A

59. 3. How often did the pain in your ***left*** hand/wrist interfere with your sleep?

Mark only one oval.

- Always
- Often
- Sometimes
- Rarely
- Never
- N/A

60. 4. How often did the pain in your ***left*** hand/wrist interfere with your daily activities (such as eating or bathing)?

Mark only one oval.

- Always
- Often
- Sometimes
- Rarely
- Never
- N/A

61. 5. How often did the pain in your ***left*** hand/wrist make you unhappy?

Mark only one oval.

- Always
- Often
- Sometimes
- Rarely
- Never
- N/A

MICHIGAN HAND OUTCOMES QUESTIONNAIRE (MHQ)

Instructions: This survey asks for your views about your hands and your health. This information will help keep track of how you feel and how well you are able to do your usual activities.

- Answer **EVERY** question, even if you do not experience any problems with the hand and/or wrist. If you are unsure about how to answer a question, please give the best answer you can.

V. A. The following questions refer to the appearance (look) of your ***right*** hand **during the past week.** (Please circle one answer for each question)

62. 1. I am satisfied with the appearance (look) of my ***right*** hand.

Mark only one oval.

- Strongly Agree
- Agree
- Neither Agree nor Disagree
- Disagree
- Strongly Disagree
- N/A

63. 2. The appearance (look) of my ***right*** hand sometimes made me uncomfortable in public.

Mark only one oval.

- Strongly Agree
- Agree
- Neither Agree nor Disagree
- Disagree
- Strongly Disagree
- N/A

64. 3. The appearance (look) of my ***right*** hand made me depressed.

Mark only one oval.

- Strongly Agree
- Agree
- Neither Agree nor Disagree
- Disagree
- Strongly Disagree
- N/A

65. 4. The appearance (look) of my ***right*** hand interfered with my normal social activities.

Mark only one oval.

- Strongly Agree
- Agree
- Neither Agree nor Disagree
- Disagree
- Strongly Disagree
- N/A

MICHIGAN HAND OUTCOMES QUESTIONNAIRE (MHQ)

Instructions: This survey asks for your views about your hands and your health. This information will help keep track of how you feel and how well you are able to do your usual activities.

- Answer **EVERY** question, even if you do not experience any problems with the hand and/or wrist. If you are unsure about how to answer a question, please give the best answer you can.

V. B. The following questions refer to the appearance (look) of your ***left*** hand **during the past week.** (Please circle one answer for each question)

66. 1. I am satisfied with the appearance (look) of my ***left*** hand.

Mark only one oval.

- Strongly Agree
- Agree
- Neither Agree nor Disagree
- Disagree
- Strongly Disagree
- N/A

67. 2. The appearance (look) of my ***left*** hand sometimes made me uncomfortable in public.

Mark only one oval.

- Strongly Agree
- Agree
- Neither Agree nor Disagree
- Disagree
- Strongly Disagree
- N/A

68. 3. The appearance (look) of my ***left*** hand made me depressed.

Mark only one oval.

- Strongly Agree
- Agree
- Neither Agree nor Disagree
- Disagree
- Strongly Disagree
- N/A

69. 4. The appearance (look) of my ***left*** hand interfered with my normal social activities.

Mark only one oval.

- Strongly Agree
- Agree
- Neither Agree nor Disagree
- Disagree
- Strongly Disagree
- N/A

MICHIGAN HAND OUTCOMES QUESTIONNAIRE (MHQ)

Instructions: This survey asks for your views about your hands and your health. This information will help keep track of how you feel and how well you are able to do your usual activities.

- Answer **EVERY** question, even if you do not experience any problems with the hand and/or wrist. If you are unsure about how to answer a question, please give the best answer you can.

VI. A. The following questions refer to your satisfaction with your **right** hand/wrist **during the past week.** (Please circle one answer for each question)

70. 1. Overall function of your ***right*** hand

Mark only one oval.

- Very Satisfied
- Somewhat Satisfied
- Neither Satisfied nor Dissatisfied
- Somewhat Dissatisfied
- Very Dissatisfied
- N/A

71. 2. Motion of the fingers in your ***right*** hand

Mark only one oval.

- Very Satisfied
- Somewhat Satisfied
- Neither Satisfied nor Dissatisfied
- Somewhat Dissatisfied
- Very Dissatisfied
- N/A

72. 3. Motion of your ***right*** wrist

Mark only one oval.

- Very Satisfied
- Somewhat Satisfied
- Neither Satisfied nor Dissatisfied
- Somewhat Dissatisfied
- Very Dissatisfied
- N/A

73. 4. Strength of your ***right*** hand

Mark only one oval.

- Very Satisfied
- Somewhat Satisfied
- Neither Satisfied nor Dissatisfied
- Somewhat Dissatisfied
- Very Dissatisfied
- N/A

74. 5. Pain level of your ***right*** hand

Mark only one oval.

- Very Satisfied
- Somewhat Satisfied
- Neither Satisfied nor Dissatisfied
- Somewhat Dissatisfied
- Very Dissatisfied
- N/A

75. 6. Sensation (feeling) of your ***right*** hand

Mark only one oval.

- Very Satisfied
- Somewhat Satisfied
- Neither Satisfied nor Dissatisfied
- Somewhat Dissatisfied
- Very Dissatisfied
- N/A

MICHIGAN HAND OUTCOMES QUESTIONNAIRE (MHQ)

Instructions: This survey asks for your views about your hands and your health. This information will help keep track of how you feel and how well you are able to do your usual activities.

- Answer **EVERY** question, even if you do not experience any problems with the hand and/or wrist. If you are unsure about how to answer a question, please give the best answer you can.

VI. B. The following questions refer to your satisfaction with your **left** hand/wrist **during the past week.** (Please circle one answer for each question)

76. 1. Overall function of your ***left*** hand

Mark only one oval.

- Very Satisfied
- Somewhat Satisfied
- Neither Satisfied nor Dissatisfied
- Somewhat Dissatisfied
- Very Dissatisfied
- N/A

77. 2. Motion of the fingers in your ***left*** hand

Mark only one oval.

- Very Satisfied
- Somewhat Satisfied
- Neither Satisfied nor Dissatisfied
- Somewhat Dissatisfied
- Very Dissatisfied
- N/A

78. 3. Motion of your ***left*** wrist

Mark only one oval.

- Very Satisfied
- Somewhat Satisfied
- Neither Satisfied nor Dissatisfied
- Somewhat Dissatisfied
- Very Dissatisfied
- N/A

79. 4. Strength of your ***left*** hand

Mark only one oval.

- Very Satisfied
- Somewhat Satisfied
- Neither Satisfied nor Dissatisfied
- Somewhat Dissatisfied
- Very Dissatisfied
- N/A

80. 5. Pain level of your ***left*** hand

Mark only one oval.

- Very Satisfied
- Somewhat Satisfied
- Neither Satisfied nor Dissatisfied
- Somewhat Dissatisfied
- Very Dissatisfied
- N/A

81. 6. Sensation (feeling) of your ***left*** hand

Mark only one oval.

- Very Satisfied
- Somewhat Satisfied
- Neither Satisfied nor Dissatisfied
- Somewhat Dissatisfied
- Very Dissatisfied
- N/A

MICHIGAN HAND OUTCOMES QUESTIONNAIRE (MHQ)

Instructions: This survey asks for your views about your hands and your health. This information will help keep track of how you feel and how well you are able to do your usual activities.

- Answer **EVERY** question, even if you do not experience any problems with the hand and/or wrist. If you are unsure about how to answer a question, please give the best answer you can.

82. Please briefly describe your injury (Location: Left vs Right (which finger or hand) | Size of Trauma | back or palm side of the hand)
(Eg. Left Index finger cut 2cm long on the palmar side)

83. 5. How many Physical Rehabilitation Sessions have you had since your surgery?

84. 6. How does your doctor/therapist report the progress of your healing?

Mark only one oval.

- Progressing ahead of schedule
- Progressing on time with schedule
- Progressing behind of schedule
- Not Sure

85. 7. Is there lawsuit action regarding your case currently pending?

Mark only one oval.

- Yes
- No
- Choose not to say

This content is neither created nor endorsed by Google.

Google Forms
