# Supplementary material for: Handy insights: Could online patient-reported outcome measures be used to assess hand injury rehabilitation?
Source: MethodsX. 2024 Nov 7;13:103029. doi: 10.1016/j.mex.2024.103029 (PMC11600655; doi:10.1016/j.mex.2024.103029)
Supplement: Supplementary file 4 [file mmc4.pdf]

Questionnaire 2

Study Title: *Assessing Patient Reported Outcomes Following Hand Trauma Surgery: A Prospective Cohort Study*

Chief Investigator:

Medical Student:
Email Address:

For your reference, please review the following: Participant Information Leaflet and Data Protection Notice

* Indicates required question

1. Email *

MICHIGAN HAND OUTCOMES QUESTIONNAIRE (MHQ)

Instructions: This survey asks for your views about your hands and your health. This information will help keep track of how you feel and how well you are able to do your usual activities.

- Answer **EVERY** question, even if you do not experience any problems with the hand and/or wrist. If you are unsure about how to answer a question, please give the best answer you can.

I. The following questions refer to the function of your hand(s)/wrist(s) ***during the past week.***

A. The following questions refer to your ***right*** hand/wrist. (Please circle one answer for each question).

2. 1. Overall, how well did your ***right*** hand work? *

Mark only one oval.

- Very Good
- Good
- Fair
- Poor
- Very Poor
- N/A

3. 2. How well did your ***right*** fingers move? *

Mark only one oval.

- Very Good
- Good
- Fair
- Poor
- Very Poor
- N/A

4. 3. How well did your ***right*** wrist move? *

Mark only one oval.

- Very Good
- Good
- Fair
- Poor
- Very Poor
- N/A

5. 4. How was the strength in your ***right*** hand? *

Mark only one oval.

- Very Good
- Good
- Fair
- Poor
- Very Poor
- N/A

6. 5. How was the sensation (feeling) in your ***right*** hand? *

Mark only one oval.

- Very Good
- Good
- Fair
- Poor
- Very Poor
- N/A

MICHIGAN HAND OUTCOMES QUESTIONNAIRE (MHQ)

Instructions: This survey asks for your views about your hands and your health. This information will help keep track of how you feel and how well you are able to do your usual activities.

- Answer **EVERY** question, even if you do not experience any problems with the hand and/or wrist. If you are unsure about how to answer a question, please give the best answer you can.

I. The following questions refer to the function of your hand(s)/wrist(s) ***during the past week.***

B. The following questions refer to your ***left*** hand/wrist. (Please circle one answer for each question).

7. 1. Overall, how well did your ***left*** hand work? *

Mark only one oval.

- Very Good
- Good
- Fair
- Poor
- Very Poor
- N/A

8. 2. How well did your ***left*** fingers move? *

Mark only one oval.

- Very Good
- Good
- Fair
- Poor
- Very Poor
- N/A

9. 3. How well did your ***left*** wrist move? *

Mark only one oval.

- Very Good
- Good
- Fair
- Poor
- Very Poor
- N/A

10. 4. How was the strength in your ***left*** hand? *

Mark only one oval.

- Very Good
- Good
- Fair
- Poor
- Very Poor
- N/A

11. 5. How was the sensation (feeling) in your ***left*** hand? *

Mark only one oval.

- Very Good
- Good
- Fair
- Poor
- Very Poor
- N/A

MICHIGAN HAND OUTCOMES QUESTIONNAIRE (MHQ)

Instructions: This survey asks for your views about your hands and your health. This information will help keep track of how you feel and how well you are able to do your usual activities.

- Answer **EVERY** question, even if you do not experience any problems with the hand and/or wrist. If you are unsure about how to answer a question, please give the best answer you can.

II. The following questions refer to the ability of your hand(s) to do certain tasks ***during the past week***. (Please circle one answer for each question). If you do not do a certain task, please estimate the difficulty with which you would have in performing it.

A. How difficult was it for you to perform the following activities using your ***right hand***?

12. 1. Turn a door knob *

Mark only one oval.

- Not at All Difficult
- A Little Difficult
- Somewhat Difficult
- Moderately Difficult
- Very Difficult
- N/A

13. 2. Pick up a coin *

Mark only one oval.

- Not at All Difficult
- A Little Difficult
- Somewhat Difficult
- Moderately Difficult
- Very Difficult
- N/A

14. 3. Hold a glass of water *

Mark only one oval.

- Not at All Difficult
- A Little Difficult
- Somewhat Difficult
- Moderately Difficult
- Very Difficult
- N/A

15. 4. Turn a key in a lock *

Mark only one oval.

- Not at All Difficult
- A Little Difficult
- Somewhat Difficult
- Moderately Difficult
- Very Difficult
- N/A

16. 5. Hold a frying pan *

Mark only one oval.

- Not at All Difficult
- A Little Difficult
- Somewhat Difficult
- Moderately Difficult
- Very Difficult
- N/A

MICHIGAN HAND OUTCOMES QUESTIONNAIRE (MHQ)

Instructions: This survey asks for your views about your hands and your health. This information will help keep track of how you feel and how well you are able to do your usual activities.

- Answer **EVERY** question, even if you do not experience any problems with the hand and/or wrist. If you are unsure about how to answer a question, please give the best answer you can.

II. The following questions refer to the ability of your hand(s) to do certain tasks ***during the past week***. (Please circle one answer for each question). If you do not do a certain task, please estimate the difficulty with which you would have in performing it.
B. How difficult was it for you to perform the following activities using your ***left hand***?

17. 1. Turn a door knob *

Mark only one oval.

- Not at All Difficult
- A Little Difficult
- Somewhat Difficult
- Moderately Difficult
- Very Difficult
- N/A

18. 2. Pick up a coin *

Mark only one oval.

- Not at All Difficult
- A Little Difficult
- Somewhat Difficult
- Moderately Difficult
- Very Difficult
- N/A

19. 3. Hold a glass of water *

Mark only one oval.

- Not at All Difficult
- A Little Difficult
- Somewhat Difficult
- Moderately Difficult
- Very Difficult
- N/A

20. 4. Turn a key in a lock *

Mark only one oval.

- Not at All Difficult
- A Little Difficult
- Somewhat Difficult
- Moderately Difficult
- Very Difficult
- N/A

21. 5. Hold a frying pan *

Mark only one oval.

- Not at All Difficult
- A Little Difficult
- Somewhat Difficult
- Moderately Difficult
- Very Difficult
- N/A

MICHIGAN HAND OUTCOMES QUESTIONNAIRE (MHQ)

Instructions: This survey asks for your views about your hands and your health. This information will help keep track of how you feel and how well you are able to do your usual activities.

- Answer **EVERY** question, even if you do not experience any problems with the hand and/or wrist. If you are unsure about how to answer a question, please give the best answer you can.

II. The following questions refer to the ability of your hand(s) to do certain tasks ***during the past week***. (Please circle one answer for each question). If you do not do a certain task, please estimate the difficulty with which you would have in performing it.
C. How difficult was it for you to perform the following activities using ***both of your hands?***

22. 1. Open a jar *

Mark only one oval.

- Not at All Difficult
- A Little Difficult
- Somewhat Difficult
- Moderately Difficult
- Very Difficult
- N/A

23. 2. Button a shirt/blouse *

Mark only one oval.

- Not at All Difficult
- A Little Difficult
- Somewhat Difficult
- Moderately Difficult
- Very Difficult
- N/A

24. 3. Eat with a knife/fork *

Mark only one oval.

- Not at All Difficult
- A Little Difficult
- Somewhat Difficult
- Moderately Difficult
- Very Difficult
- N/A

25. 4. Carry a grocery bag *

Mark only one oval.

- Not at All Difficult
- A Little Difficult
- Somewhat Difficult
- Moderately Difficult
- Very Difficult
- N/A

26. 5. Wash dishes *

Mark only one oval.

- Not at All Difficult
- A Little Difficult
- Somewhat Difficult
- Moderately Difficult
- Very Difficult
- N/A

27. 6. Wash your hair *

Mark only one oval.

- Not at All Difficult
- A Little Difficult
- Somewhat Difficult
- Moderately Difficult
- Very Difficult
- N/A

28. 7. Tie shoelaces/knots *

Mark only one oval.

- Not at All Difficult
- A Little Difficult
- Somewhat Difficult
- Moderately Difficult
- Very Difficult
- N/A

MICHIGAN HAND OUTCOMES QUESTIONNAIRE (MHQ)

Instructions: This survey asks for your views about your hands and your health. This information will help keep track of how you feel and how well you are able to do your usual activities.

- Answer **EVERY** question, even if you do not experience any problems with the hand and/or wrist. If you are unsure about how to answer a question, please give the best answer you can.

III. The following questions refer to how you did in your ***normal work*** (including both housework and school work) during the ***past four weeks.*** (Please circle one answer for each question).

29. 1. How often were you unable to do your work because of problems with your hand(s)/wrist(s)? *

Mark only one oval.

- Always
- Often
- Sometimes
- Rarely
- Never
- N/A

30. 2. How often did you have to shorten your work day because of problems with your hand(s)/ wrist(s)? *

Mark only one oval.

- Always
- Often
- Sometimes
- Rarely
- Never
- N/A

31. 3. How often did you have to take it easy at your work because of problems with your hand(s)/ wrist(s)? *

Mark only one oval.

- Always
- Often
- Sometimes
- Rarely
- Never
- N/A

32. 4. How often did you accomplish less in your work because of problems with your hand(s)/ wrist(s)? *

Mark only one oval.

- Always
- Often
- Sometimes
- Rarely
- Never
- N/A

33. 5. How often did you take longer to do the tasks in your work because of problems with your hand(s)/ wrist(s)? *

Mark only one oval.

- Always
- Often
- Sometimes
- Rarely
- Never
- N/A

MICHIGAN HAND OUTCOMES QUESTIONNAIRE (MHQ)

Instructions: This survey asks for your views about your hands and your health. This information will help keep track of how you feel and how well you are able to do your usual activities.

- Answer **EVERY** question, even if you do not experience any problems with the hand and/or wrist. If you are unsure about how to answer a question, please give the best answer you can.

IV. The following questions refer to how much ***pain*** you had in your hand(s)/wrist(s) ***during the past week.*** (Please circle one answer for each question).
A. The following questions refer to **pain** in your **right** hand/wrist.

34. 1. How often did you have pain in your ***right*** hand/wrist? *

Mark only one oval.

- Always
- Often
- Sometimes
- Rarely
- Never *Skip to question 40*

MICHIGAN HAND OUTCOMES QUESTIONNAIRE (MHQ)

Instructions: This survey asks for your views about your hands and your health. This information will help keep track of how you feel and how well you are able to do your usual activities.

- Answer **EVERY** question, even if you do not experience any problems with the hand and/or wrist. If you are unsure about how to answer a question, please give the best answer you can.

IV. The following questions refer to how much ***pain*** you had in your hand(s)/wrist(s) ***during the past week.*** (Please circle one answer for each question).
A. The following questions refer to **pain** in your **right** hand/wrist.

35. 2. Please describe the pain you had in your ***right*** hand/wrist *

Mark only one oval.

- Very Mild
- Mild
- Moderate
- Severe
- Very Severe
- N/A

36. 3. How often did the pain in your ***right*** hand/wrist interfere with your sleep? *

Mark only one oval.

- Always
- Often
- Sometimes
- Rarely
- Never
- N/A

37. 4. How often did the pain in your ***right*** hand/wrist interfere with your daily activities (such as eating or bathing)? *

Mark only one oval.

- Always
- Often
- Sometimes
- Rarely
- Never
- N/A

38. 5. How often did the pain in your ***right*** hand/wrist make you unhappy? *

Mark only one oval.

- Always
- Often
- Sometimes
- Rarely
- Never
- N/A

MICHIGAN HAND OUTCOMES QUESTIONNAIRE (MHQ)

Instructions: This survey asks for your views about your hands and your health. This information will help keep track of how you feel and how well you are able to do your usual activities.

- Answer **EVERY** question, even if you do not experience any problems with the hand and/or wrist. If you are unsure about how to answer a question, please give the best answer you can.

IV. The following questions refer to how much ***pain*** you had in your hand(s)/wrist(s) ***during the past week.*** (Please circle one answer for each question).
B. The following questions refer to **pain** in your **left** hand/wrist.

39. 1. How often did you have pain in your ***left*** hand/wrist? *

Mark only one oval.

- Always
- Often
- Sometimes
- Rarely
- Never *Skip to question 44*

MICHIGAN HAND OUTCOMES QUESTIONNAIRE (MHQ)

Instructions: This survey asks for your views about your hands and your health. This information will help keep track of how you feel and how well you are able to do your usual activities.

- Answer **EVERY** question, even if you do not experience any problems with the hand and/or wrist. If you are unsure about how to answer a question, please give the best answer you can.

40. 2. Please describe the pain you had in your ***left*** hand/wrist *

Mark only one oval.

- Very Mild
- Mild
- Moderate
- Severe
- Very Severe
- N/A

41. 3. How often did the pain in your ***left*** hand/wrist interfere with your sleep? *

Mark only one oval.

- Always
- Often
- Sometimes
- Rarely
- Never
- N/A

42. 4. How often did the pain in your ***left*** hand/wrist interfere with your daily activities (such as eating or bathing)? *

Mark only one oval.

- Always
- Often
- Sometimes
- Rarely
- Never
- N/A

43. 5. How often did the pain in your ***left*** hand/wrist make you unhappy? *

Mark only one oval.

- Always
- Often
- Sometimes
- Rarely
- Never
- N/A

MICHIGAN HAND OUTCOMES QUESTIONNAIRE (MHQ)

Instructions: This survey asks for your views about your hands and your health. This information will help keep track of how you feel and how well you are able to do your usual activities.

- Answer **EVERY** question, even if you do not experience any problems with the hand and/or wrist. If you are unsure about how to answer a question, please give the best answer you can.

V. A. The following questions refer to the appearance (look) of your ***right*** hand **during the past week.** (Please circle one answer for each question)

44. 1. I am satisfied with the appearance (look) of my ***right*** hand. *

Mark only one oval.

- Strongly Agree
- Agree
- Neither Agree nor Disagree
- Disagree
- Strongly Disagree
- N/A

45. 2. The appearance (look) of my ***right*** hand sometimes made me uncomfortable in public. *

Mark only one oval.

- Strongly Agree
- Agree
- Neither Agree nor Disagree
- Disagree
- Strongly Disagree
- N/A

46. 3. The appearance (look) of my ***right*** hand made me depressed. *

Mark only one oval.

- Strongly Agree
- Agree
- Neither Agree nor Disagree
- Disagree
- Strongly Disagree
- N/A

47. 4. The appearance (look) of my ***right*** hand interfered with my normal social activities. *

Mark only one oval.

- Strongly Agree
- Agree
- Neither Agree nor Disagree
- Disagree
- Strongly Disagree
- N/A

MICHIGAN HAND OUTCOMES QUESTIONNAIRE (MHQ)

Instructions: This survey asks for your views about your hands and your health. This information will help keep track of how you feel and how well you are able to do your usual activities.

- Answer **EVERY** question, even if you do not experience any problems with the hand and/or wrist. If you are unsure about how to answer a question, please give the best answer you can.

V. B. The following questions refer to the appearance (look) of your ***left*** hand **during the past week.** (Please circle one answer for each question)

48. 1. I am satisfied with the appearance (look) of my ***left*** hand. *

Mark only one oval.

- Strongly Agree
- Agree
- Neither Agree nor Disagree
- Disagree
- Strongly Disagree
- N/A

49. 2. The appearance (look) of my ***left*** hand sometimes made me uncomfortable in public. *

Mark only one oval.

- Strongly Agree
- Agree
- Neither Agree nor Disagree
- Disagree
- Strongly Disagree
- N/A

50. 3. The appearance (look) of my ***left*** hand made me depressed. *

Mark only one oval.

- Strongly Agree
- Agree
- Neither Agree nor Disagree
- Disagree
- Strongly Disagree
- N/A

51. 4. The appearance (look) of my ***left*** hand interfered with my normal social activities. *

Mark only one oval.

- Strongly Agree
- Agree
- Neither Agree nor Disagree
- Disagree
- Strongly Disagree
- N/A

MICHIGAN HAND OUTCOMES QUESTIONNAIRE (MHQ)

Instructions: This survey asks for your views about your hands and your health. This information will help keep track of how you feel and how well you are able to do your usual activities.

- Answer **EVERY** question, even if you do not experience any problems with the hand and/or wrist. If you are unsure about how to answer a question, please give the best answer you can.

VI. A. The following questions refer to your satisfaction with your **right** hand/wrist **during the past week.** (Please circle one answer for each question)

52. 1. Overall function of your ***right*** hand *

Mark only one oval.

- Very Satisfied
- Somewhat Satisfied
- Neither Satisfied nor Dissatisfied
- Somewhat Dissatisfied
- Very Dissatisfied
- N/A

53. 2. Motion of the fingers in your ***right*** hand *

Mark only one oval.

- Very Satisfied
- Somewhat Satisfied
- Neither Satisfied nor Dissatisfied
- Somewhat Dissatisfied
- Very Dissatisfied
- N/A

54. 3. Motion of your ***right*** wrist *

Mark only one oval.

- Very Satisfied
- Somewhat Satisfied
- Neither Satisfied nor Dissatisfied
- Somewhat Dissatisfied
- Very Dissatisfied
- N/A

55. 4. Strength of your ***right*** hand *

Mark only one oval.

- Very Satisfied
- Somewhat Satisfied
- Neither Satisfied nor Dissatisfied
- Somewhat Dissatisfied
- Very Dissatisfied
- N/A

56. 5. Pain level of your ***right*** hand *

Mark only one oval.

- Very Satisfied
- Somewhat Satisfied
- Neither Satisfied nor Dissatisfied
- Somewhat Dissatisfied
- Very Dissatisfied
- N/A

57. 6. Sensation (feeling) of your ***right*** hand *

Mark only one oval.

- Very Satisfied
- Somewhat Satisfied
- Neither Satisfied nor Dissatisfied
- Somewhat Dissatisfied
- Very Dissatisfied
- N/A

MICHIGAN HAND OUTCOMES QUESTIONNAIRE (MHQ)

Instructions: This survey asks for your views about your hands and your health. This information will help keep track of how you feel and how well you are able to do your usual activities.

- Answer **EVERY** question, even if you do not experience any problems with the hand and/or wrist. If you are unsure about how to answer a question, please give the best answer you can.

VI. B. The following questions refer to your satisfaction with your **left** hand/wrist **during the past week.** (Please circle one answer for each question)

58. 1. Overall function of your ***left*** hand *

Mark only one oval.

- Very Satisfied
- Somewhat Satisfied
- Neither Satisfied nor Dissatisfied
- Somewhat Dissatisfied
- Very Dissatisfied
- N/A

59. 2. Motion of the fingers in your ***left*** hand *

Mark only one oval.

- Very Satisfied
- Somewhat Satisfied
- Neither Satisfied nor Dissatisfied
- Somewhat Dissatisfied
- Very Dissatisfied
- N/A

60. 3. Motion of your ***left*** wrist *

Mark only one oval.

- Very Satisfied
- Somewhat Satisfied
- Neither Satisfied nor Dissatisfied
- Somewhat Dissatisfied
- Very Dissatisfied
- N/A

61. 4. Strength of your ***left*** hand *

Mark only one oval.

- Very Satisfied
- Somewhat Satisfied
- Neither Satisfied nor Dissatisfied
- Somewhat Dissatisfied
- Very Dissatisfied
- N/A

62. 5. Pain level of your ***left*** hand *

Mark only one oval.

- Very Satisfied
- Somewhat Satisfied
- Neither Satisfied nor Dissatisfied
- Somewhat Dissatisfied
- Very Dissatisfied
- N/A

63. 6. Sensation (feeling) of your ***left*** hand *

Mark only one oval.

- Very Satisfied
- Somewhat Satisfied
- Neither Satisfied nor Dissatisfied
- Somewhat Dissatisfied
- Very Dissatisfied
- N/A

MICHIGAN HAND OUTCOMES QUESTIONNAIRE (MHQ)

Instructions: This survey asks for your views about your hands and your health. This information will help keep track of how you feel and how well you are able to do your usual activities.

- Answer **EVERY** question, even if you do not experience any problems with the hand and/or wrist. If you are unsure about how to answer a question, please give the best answer you can.

Please provide the following information about yourself. (Please circle one answer for each question).

64. 1. a. Please describe the type of job you did **before** you injured your hands(s). *

____

65. 2. b. Please describe the type of job you are doing **now**. *

____

66. 3. How many days after your surgery did you return to work/expected to start work? *

____

67. 4. How long after your surgery did you return to the same job you were doing before your injury? *

____

68. 5. How many Physical Rehabilitation Sessions have you had since your surgery? *

____

69. 6. How does your doctor/therapist report the progress of your healing? *

Mark only one oval.

- Progressing ahead of schedule
- Progressing on time with schedule
- Progressing behind of schedule
- Not Sure

70. 7. Is there lawsuit action regarding your case currently pending? *

Mark only one oval.

- Yes
- No
- Choose not to say

This content is neither created nor endorsed by Google.

Google Forms
