# Supplementary material for: Handy insights: Could online patient-reported outcome measures be used to assess hand injury rehabilitation?
Source: MethodsX. 2024 Nov 7;13:103029. doi: 10.1016/j.mex.2024.103029 (PMC11600655; doi:10.1016/j.mex.2024.103029)
Supplement: Supplementary file 7 [file mmc7.pdf]

HAND INJURY SEVERITY SCORE

Campbell DA, Kay SPJ. The Hand Injury Severity Scoring System. J Hand Surg. 1996 Jun 1;21(3):295–8.

Patient Details

- **Name (First Last):**
- **MRN:**

***Was Patient Consent Obtained? Yes / No**

HAND			
Absolut Values (Hand)	Dorsum	<1cm2	
		>1cm2	
		>5cm2	
	Palm	<1cm2	
		>1cm2	
		>5cm2	
Absolut Values	Reccurent Branch Median Nerve		
	Deep Branch Ulnar Nerve		

THUMB			
INTEGUMENT			
Skin Loss	Digits	Dorsum	<1cm2
			>1cm2
		Volar	<25%
			>25%
Skin Laceration			<1cm
			> 1cm
Nail damage			
SKELETAL			
Fractures	Simple shaft		
	Comminuted shaft		
	Inter-articular IP J		
	Inter-articular MCP J		
Dislocations	Open		
	closed		
Ligament Injury	sprain		
	rupture		
MOTOR			
Extensor Tendon	Proximal to PIP J		
	Distal to PIP J		
Flexor Profundus	Zone 1		
	Zone 2		
	Zone 3		
Flexor Superficialis			
Intrinsics			
NEURAL			
Weighted Values	Digital Nerve x 1		
	Digital Nerve x 2		

INDEX			
INTEGUMENT			
Skin Loss	Digits	Dorsum	<1cm2
			>1cm2
		Volar	<25%
			>25%
Skin Laceration			<1cm
			> 1cm
Nail damage			
SKELETAL			
Fractures	Simple shaft		
	Comminuted shaft		
	Inter-articular DIP J		
	Inter-articular PIP/MIP J		
	Inter-articular MCP J		
Dislocations	Open		
	closed		
Ligament Injury	sprain		
	rupture		
MOTOR			
Extensor Tendon	Proximal to PIP J		
	Distal to PIP J		
Flexor Profundus	Zone 1		
	Zone 2		
	Zone 3		
Flexor Superficialis			
Intrinsics			
NEURAL			
Weighted Values	Digital Nerve x 1		
	Digital Nerve x 2		

MIDDLE			
INTEGUMENT			
Skin Loss	Digits	Dorsum	<1cm2
			>1cm2
		Volar	<25%
			>25%
Skin Laceration			<1cm
			> 1cm
Nail damage			
SKELETAL			
Fractures	Simple shaft		
	Comminuted shaft		
	Inter-articular DIP J		
	Inter-articular PIP/MIP J		
	Inter-articular MCP J		
Dislocations	Open		
	closed		
Ligament Injury	sprain		
	rupture		
MOTOR			
Extensor Tendon	Proximal to PIP J		
	Distal to PIP J		
Flexor Profundus	Zone 1		
	Zone 2		
	Zone 3		
Flexor Superficialis			
Intrinsics			
NEURAL			
Weighted Values	Digital Nerve x 1		
	Digital Nerve x 2		

RING			
INTEGUMENT			
Skin Loss	Digits	Dorsum	<1cm2
			>1cm2
		Volar	<25%
			>25%
Skin Laceration			<1cm
			> 1cm
Nail damage			
SKELETAL			
Fractures	Simple shaft		
	Comminuted shaft		
	Inter-articular DIP J		
	Inter-articular PIP/MIP J		
	Inter-articular MCP J		
Dislocations	Open		
	closed		
Ligament Injury	sprain		
	rupture		
MOTOR			
Extensor Tendon	Proximal to PIP J		
	Distal to PIP J		
Flexor Profundus	Zone 1		
	Zone 2		
	Zone 3		
Flexor Superficialis			
Intrinsics			
NEURAL			
Weighted Values	Digital Nerve x 1		
	Digital Nerve x 2		

LITTLE			
INTEGUMENT			
Skin Loss	Digits	Dorsum	<1cm2
			>1cm2
		Volar	<25%
			>25%
Skin Laceration			<1cm
			> 1cm
Nail damage			
SKELETAL			
Fractures	Simple shaft		
	Comminuted shaft		
	Inter-articular DIP J		
	Inter-articular PIP/MIP J		
	Inter-articular MCP J		
Dislocations	Open		
	closed		
Ligament Injury	sprain		
	rupture		
MOTOR			
Extensor Tendon	Proximal to PIP J		
	Distal to PIP J		
Flexor Profundus	Zone 1		
	Zone 2		
	Zone 3		
Flexor Superficialis			
Intrinsics			
NEURAL			
Weighted Values	Digital Nerve x 1		
	Digital Nerve x 2		
