# Supplementary material for: Handy insights: Could online patient-reported outcome measures be used to assess hand injury rehabilitation?
Source: MethodsX. 2024 Nov 7;13:103029. doi: 10.1016/j.mex.2024.103029 (PMC11600655; doi:10.1016/j.mex.2024.103029)
Supplement: Supplementary file 8 [file mmc8.pdf]

INNOVATION PARTNER
UNIVERSITY OF MICHIGAN

Order Details

- **Order #23992 Summary**
- **Contact Information**

  Sten Kajitani

- **Order Summary**

**3372 - Michigan Hand Outcomes Questionnaire (MHQ) - Academic and Research Education Use License**	$0.00
Quantity	1
Subtotal	$0.00
Shipping	–
Total	$0.00

- **Shipping Address**

  Sten Kajitani
  AH-A403C Nido Ashlin House Bandon Road
  Cork, Cork T12FX0D
  IE
  +353 873891511

  Shipping Method

  Please provide licensee information, including organization name. Product is available via download.

- **Order Components**

  1. 3372 - Michigan Hand Questionnaire 01052023- ZIP (Downloadable)

Downloads

You can click below to download your file.

get_app 3372 - Michigan Hand Questionnaire 01052023- ZIP

Help & Support

If you have any questions regarding your order, please contact:

Drew Bennett
andbenne@umich.edu
248-464-1128
