# Supplementary material for: Handy insights: Could online patient-reported outcome measures be used to assess hand injury rehabilitation?
Source: MethodsX. 2024 Nov 7;13:103029. doi: 10.1016/j.mex.2024.103029 (PMC11600655; doi:10.1016/j.mex.2024.103029)
Supplement: Supplementary file 9 [file mmc9.docx]

**Appendix 1: Hand Injury Severity Score**

[HISS PRINT.pdf](https://drive.google.com/file/d/1elMFT_CEQoJYNyt2lZpgSCB7prKNCCyc/view?usp=share_link)

**Appendix 2: Hand Injury Severity Score Calculator**

[Copy of !Hand Injury Severity Scoring Calculator](https://docs.google.com/spreadsheets/d/1d0JlLJTHFVOeBARyJHchdRNhe-iNHQXYLbCfR9BXZI8/edit?usp=sharing)

**Appendix 3: Baseline Questionnaire and MHQ Day 0 Post-op**

<https://docs.google.com/forms/d/e/1FAIpQLSfDk3PtaJI_5JP_h7ChbQE5uZ6-X9LblGeSC4l3JiNBmXN3Zg/viewform?usp=sf_link>

If you would like permission to edit, please complete page 1 of the Baseline Questionnaire in Appendix 3.

**Appendix 4: MHQ License for Use**

[Michigan Hand Outcomes Questionnaire (MHQ) - Academic and Research Education Use License.pdf](https://drive.google.com/file/d/16i14OEzuGRHYtOfznHOgTjcrzWtFBTmC/view?usp=sharing)

**Appendix 5: MHQ 2 (1 Month Post-op)**

<https://docs.google.com/forms/d/e/1FAIpQLSeWouMEOX6sVHfPZam9YLJ8NLRLxgvSupUjz_aQhn-wByuJEA/viewform?usp=sf_link>

If you would like permission to edit, please complete page 1 of the Baseline Questionnaire in Appendix 3.

**Appendix 6: MHQ 3 (2 Month Post-op)**

<https://docs.google.com/forms/d/e/1FAIpQLScm9uM5tNFG4i-po9uNgMMwV7btJ0ta5Oqpn2e6iGxPs2UcTQ/viewform?usp=sf_link>

If you would like permission to edit, please complete page 1 of the Baseline Questionnaire in Appendix 3.

**Appendix 7: MHQ 4 (3 Month Post-op)**

<https://docs.google.com/forms/d/e/1FAIpQLScmnfAnnabsj-WOKXlmnmbliQByO2Vvoj8XZMzYF7ZROOgp8w/viewform?usp=sf_link>

If you would like permission to edit, please complete page 1 of the Baseline Questionnaire in Appendix 3.
